# Structured illumination microscopy combined with machine learning enables the high throughput analysis and classification of virus structure

**DOI:** 10.7554/eLife.40183

**Published:** 2018-12-13

**Authors:** Romain F Laine, Gemma Goodfellow, Laurence J Young, Jon Travers, Danielle Carroll, Oliver Dibben, Helen Bright, Clemens F Kaminski

**Affiliations:** 1 Department of Chemical Engineering and BiotechnologyUniversity of CambridgeCambridgeUnited Kingdom; 2 MedImmune LtdCambridgeUnited Kingdom; 3 Flu-BPDMedImmuneLiverpoolUnited Kingdom; University of WollongongAustralia; Massachusetts Institute of TechnologyUnited States

**Keywords:** Newcastle disease virus, structural analysis, super-resolution microscopy, Influenza, machine learning, Virus

## Abstract

Optical super-resolution microscopy techniques enable high molecular specificity with high spatial resolution and constitute a set of powerful tools in the investigation of the structure of supramolecular assemblies such as viruses. Here, we report on a new methodology which combines Structured Illumination Microscopy (SIM) with machine learning algorithms to image and classify the structure of large populations of biopharmaceutical viruses with high resolution. The method offers information on virus morphology that can ultimately be linked with functional performance. We demonstrate the approach on viruses produced for oncolytic viriotherapy (Newcastle Disease Virus) and vaccine development (Influenza). This unique tool enables the rapid assessment of the quality of viral production with high throughput obviating the need for traditional batch testing methods which are complex and time consuming. We show that our method also works on non-purified samples from pooled harvest fluids directly from the production line.

## eLife digest

Viruses are like the Trojan horses of the biological world; they sneak their genetic code into a living cell and then hijack it, forcing that cell to produce their own viral proteins. Yet, if scientists replace the harmful genes in a virus with other genes, the virus can be transformed into a powerful tool for biology and medical science. For example, viruses can be turned into vaccines that prime the immune system to ward off future infections. Viruses could also be made to deliver the genetic code needed to repair faulty cells, and thus treat the cause of an illness from inside the body. Nevertheless, it is complicated to produce viruses like these on a large scale.

The individual viruses in one batch can be very different shapes and sizes; they can also end up displaying different proteins on their outer surface – which is the part of the virus that our immune system will see first. To optimise the production of standardised viruses, scientists need a way to test the viruses throughout the manufacture process. At the moment, the best way to do this would be with electron microscopes. Yet these microscopes cannot tell exactly which proteins are in the outer surface of the virus. Also, these methods often need purified samples of virus, so cannot be used to look at the viruses until the final stage of production.

Laine et al. now report a method that can test virus production at every step of the process. This new method uses a different type of microscopy called super-resolution imaging, which is quicker than electron microscopy and more able to deal with impurities, but can still see objects that are 500 times smaller than the width of a human hair. First, Laine et al. took pictures of many viruses with this new imaging technique, sorted the images into groups based on their appearance, and then trained computer algorithms with the pre-sorted groups (a technique called “supervised learning”). Next, the trained algorithms were shown new images of viruses and asked to classify them. The algorithms could separate images of a mixed population of viruses into six groups according to their shape and size, and then analyse each group in a specific way. For example, they would measure and report the length of filament-shaped viruses, the radius of spherical viruses and the length and width of rod-shaped viruses.

The first set of test images were of Newcastle Disease Virus, which is currently under development as a treatment for cancer. But further testing revealed that the algorithm also works for the influenza virus, which is used to make flu vaccines. The algorithm could classify the viruses in pure and impure samples, and the imaging technique could handle over 200 viruses each second.

This approach of combining super-resolution imaging with artificial intelligence could help scientists to understand what makes good vaccines and how best to optimise the production of viruses for medical purposes. It could also allow researchers to respond more rapidly to outbreaks of viral infections. The next step is to build this work into a system that can be used by the pharmaceutical industry.

## Introduction

The potential of super-resolution microscopy (SRM) to unravel details of the structure and replication of viruses was recognised early on in the development of the methodology (Betzig et al., 2006; Müller and Heilemann, 2013). Since then, SRM has been used to provide unprecedented insights into viral protein architecture (Laine et al., 2015; Zhang et al., 2015; Gray et al., 2016; Albecka et al., 2016). Previous work has focused on those SRM techniques that achieve the highest theoretical resolution, such as Stimulated Emission Depletion (STED) (Hell and Wichmann, 1994) and Single Molecule Localisation Microscopy (SMLM) (Rust et al., 2006; Heilemann et al., 2008). Whilst offering high fidelity data, the downside is the associated long acquisition time required by these methods, limiting their application to the imaging of static samples at low throughput. A much faster technique, although inferior in spatial resolution, is Structured Illumination Microscopy (SIM) (Gustafsson, 2000; Heintzmann and Cremer, 1999) and this has been applied to study large viruses such as the prototypic poxvirus (Gray et al., 2016; Horsington et al., 2012). In addition to understanding the structure of viruses, there is also a need to identify and analyse classes of structures within large viral populations, especially in the biotechnology industry where virus quality is often compromised by large scale production operations and the virus product is often characterised by significant morphological heterogeneities. In particular, campaigns of influenza immunization rely heavily on the timely and efficient production of specific virus strains. Similarly, a deeper understanding of the structural heterogeneity of oncolytic viruses such as Newcastle Disease Virus (NDV) (Ganar et al., 2014; Lichty et al., 2014) would enable optimization of the production processes and in turn improve the development of viriotherapy. However, quantifying and understanding this structural heterogeneity and relating it to virus efficacy requires the imaging of large numbers of viruses at sufficient spatial resolution to reveal characteristic morphological details. Typically, this is achieved by extracting batches from the production process, with elaborate subsequent purification and preparation steps before characterisation by Transmission Electron Microscopy (TEM) (Gad, 2007; Goldsmith and Miller, 2009; Brenner and Horne, 1959). Although TEM can achieve relatively high imaging throughput if the highest resolution is not necessary, its contrast remains unspecific and therefore does not typically permit discerning the presence of particular proteins in the virus envelope. Also, the typical signal-to-noise ratios achieved by TEM are not sufficient to permit automated, robust and efficient downstream analysis of structural features at the single particle level. It is therefore challenging for TEM to be of practical use during production operations.

Here, we demonstrate that rapid high resolution imaging with Total Internal Reflection SIM fluorescence microscopy (TIRF-SIM) (Shao et al., 2011; Kner et al., 2009; Young et al., 2016), combined with a machine learning (ML) approach to analyse and classify structures in virus batches offer a great opportunity to circumvent these problems. We present MiLeSIM (Machine Learning Structured Illumination Microscopy) as an efficient combination of SRM, ML-based classification (Van Valen et al., 2016; Sommer et al., 2011) and advanced image analysis for the quantification of morphological heterogeneities in large virus populations. We use ML algorithms to perform a classification of super-resolved images of a heterogeneous virus population into particle classes with distinct and characteristic structural features (e.g. spherical, filamentous). The classified subpopulations are then further analysed through image analysis pipelines that are specifically adapted for each structural class. We and others have shown that appropriate model fitting can lead to precision in structural parameters beyond the resolution of the images used (Laine et al., 2015; Manetsberger et al., 2015). The method combines speed and specificity and allows an in-depth exploration of large virus populations that is unachievable by TEM. The method has potentials in the industrial production of viruses, for example for oncolytic viriotherapy and vaccine development.

First, we compare TIRF-SIM with alternative imaging modalities and show that it is the method of choice to investigate virus structure at high-throughput (~220 virus particles/s, see Supplementary Note 1) with a spatial resolution reaching ~90 nm. The large datasets obtained with TIRF-SIM are then fed into an ML algorithm for the automated classification of Newcastle Disease Virus (NDV) and live attenuated influenza virus (LAIV) vaccines, enabling further shape-specific quantitative analyses for a structural description of viral subpopulations. The purpose of our study is to validate the MiLeSIM approach as a powerful analysis tool for biotechnological processes involving virus production both in industry and in the research laboratory.

## Results

### TIRF-SIM offers an optimal combination of throughput and resolution for the imaging of virus structure

**Figure 1.**
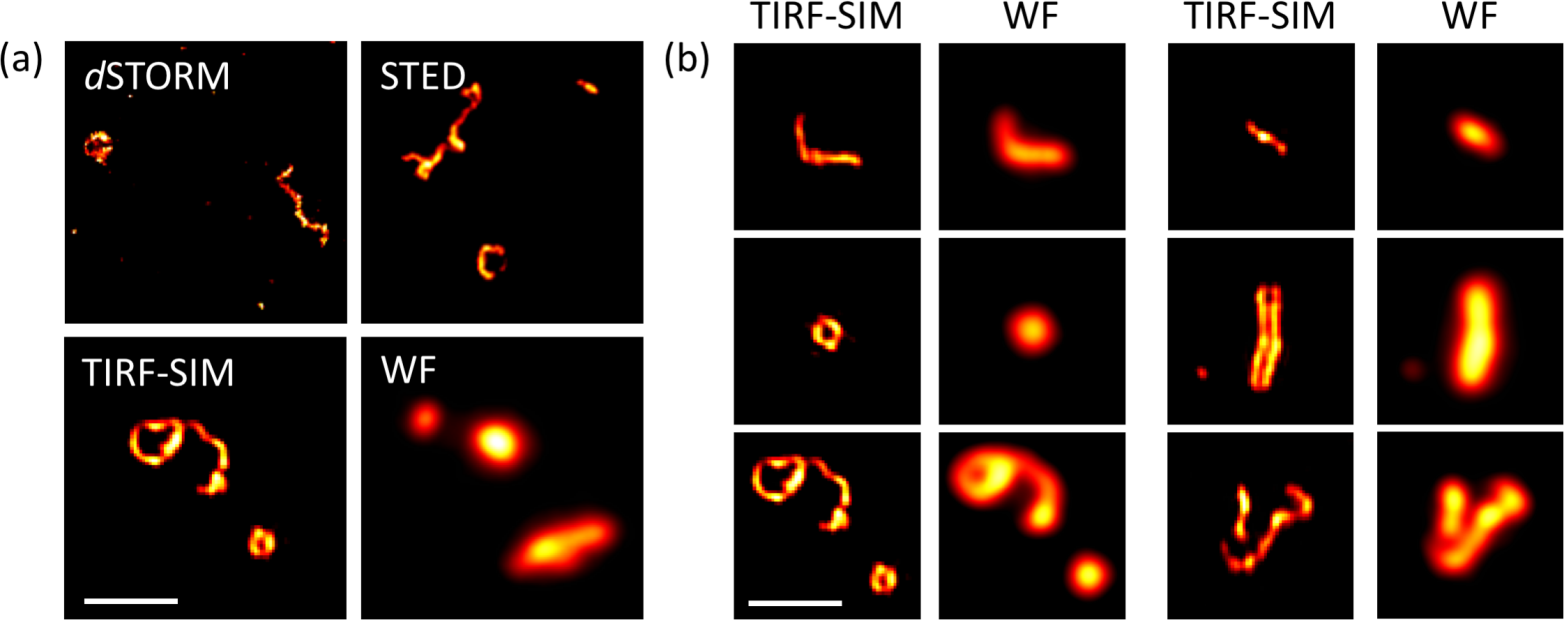
Super-resolution microscopy (SRM) for the study of NDV virus structure. (**a**) Representative images of purified NDV viruses with different imaging modalities. (**b**) Representative images of a purified NDV virus population imaged with TIRF-SIM and their corresponding TIRF wide-field image. WF: wide-field TIRF microscopy. Scale bar: 1 µm.

First, we explored and compared three common SRM modalities for the structural investigation of purified NDV virus, namely direct stochastic optical reconstruction microscopy, *d*STORM, stimulated emission depletion microscopy, STED and TIRF-SIM. NDV viruses were labelled for the envelope glycoprotein Hemagglutinin-Neuraminidase (HN) and imaged with all three SRM imaging techniques (see Figure 1). Labelling for HN allows us to directly and specifically observe the shape of the virus particles. For comparison, a conventional (non-super-resolved) TIRF wide-field image is also shown. Typical shapes observed with TIRF-SIM are shown in Figure 1(b). TIRF-SIM provides clear structural details to discern filamentous, spherical and rod-like structures in large NDV populations. A comparison of performance parameters (resolution and imaging speed) for the different methods is presented in Figure 1—figure supplement 1(a). It is clear that improving resolution beyond the ~90 nm offered by TIRF-SIM (see Figure 1—figure supplement 1(b)) comes at a significant cost in acquisition times and throughput. Furthermore, although *d*STORM and STED offer theoretically higher resolution than SIM, the images obtained with these methods do not reveal additional structural details that are not also resolved by TIRF-SIM images. This indicates that the ~2 fold resolution improvement provided by TIRF-SIM is sufficient for the structural study presented here.

**Figure 1—figure supplement 1.**
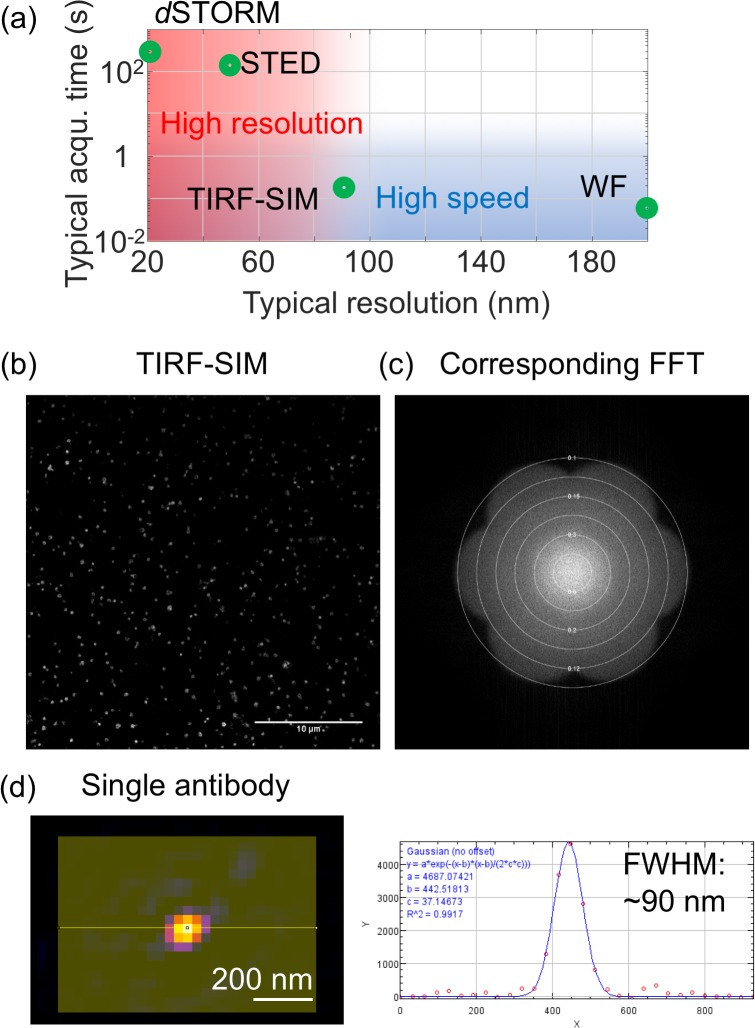
Resolution in SRM. (**a**) Typical spatial resolution and acquisition times for the imaging of NDV structures highlighting the trade-off between speed and resolution (**b**) Representative TIRF-SIM image obtained from purified B Victoria LAIV and its corresponding Fourier transform (**c**). The Fourier transform highlights the resolution ~90 nm. The plot was obtained using the SIMcheck plugin (Ball et al., 2015). (**d**) Image and cross section of a single secondary antibody labelled with DyLight 488, showing a FWHM of ~90 nm. FFT: Fast Fourier transform.

**Figure 1—figure supplement 2.**
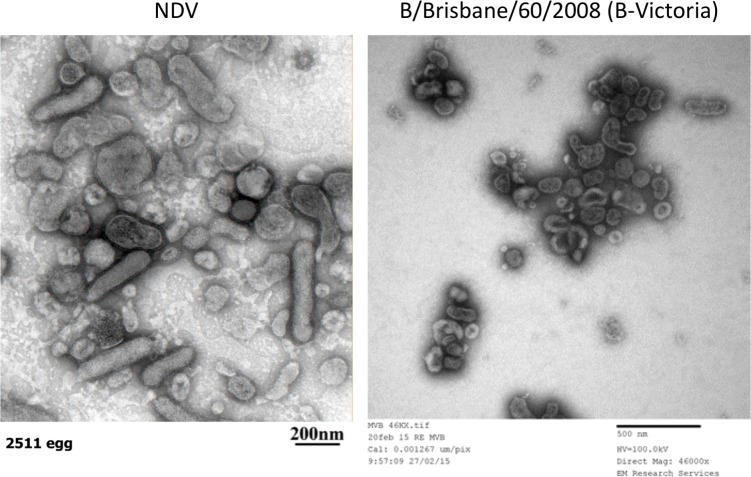
Electron micrographs of NDV and B-Victoria viruses. These images were obtained from a Philips CM 100 Compustage (FEI) TEM and negative staining.

Traditionally, EM has been the method of choice for observing sub-diffraction structures of virus particles (see Figure 1—figure supplement 2 for examples of particles). Here we show that TIRF-SIM can offer significant advantages compared to EM (summarized in Table 1). The improvement in molecular specificity allows an unambiguous identification of viral components; the high signal-to-noise ratio (SNR) furthermore enables a robust and straightforward application of further image analysis steps (identification and classification of virus particles). Also, the capability of investigating unpurified and aqueous samples makes TIRF-SIM ideally suited to the present application.

**Table 1.** Comparison of the key performance parameters of TIRF-SIM (proposed method) and EM in the context of high throughput imaging of virus structure. The resolution and acquisition time of EM were quoted for a standard TEM imaging (Philips CM 100 Compustage (FEI) Transmission Electron Microscope with an AMT CCD camera). *for a comparable field-of-view.

	**Tirf-sim**	**EM**
Contrast	Fluorescence	Electron scattering
Molecular specificity	Very high	Medium to low
Spatial resolution achievable	~90 nm	~1 Å
Acquisition time/1000 virus particles*	2 s	2 s
Typical field of view size	30 µm x 30 µm	500 nm x 500 nm
Sample preparation complexity	Low	Low to Medium
Compatibility with aqueous buffers	High	Low
Compatibility with non-purified samples	High	Low
Signal to noise ratio achievable	Very high	Medium
Sample preparation time	Low (2–3 hr)	Low to High
Expertise required for imaging	Medium	Medium
Cost	Low (£100 k)	Medium (£250 k)

### Workflow of MiLeSIM

**Figure 2.**
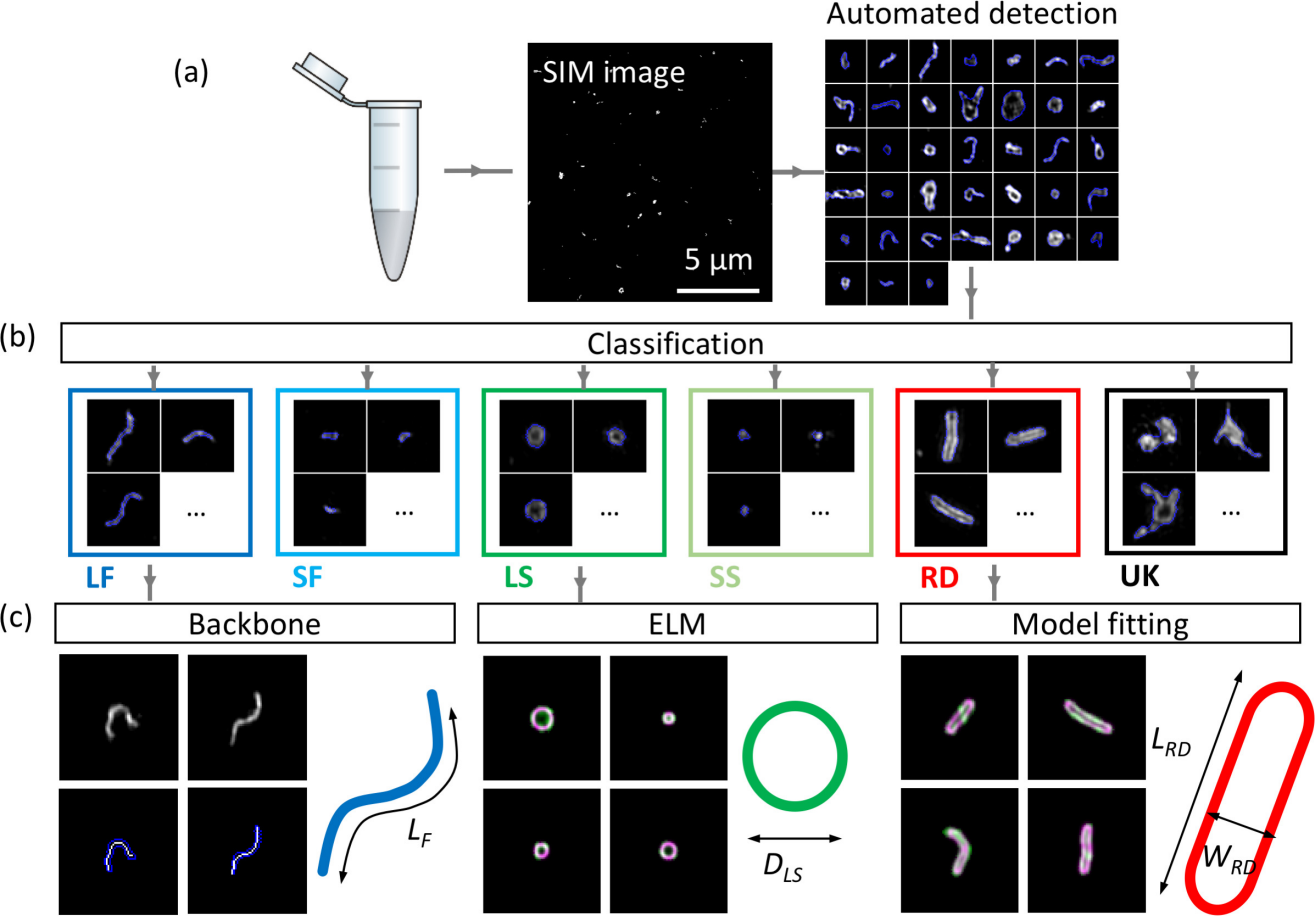
Workflow of automated detection, classification and analysis of NDV viral particles. SIM image (**a**) and segmented particles (**b**). The classified single-virus images (**b**) can be further analysed with a set of class-specific tools (**c**). For the backbone analysis the mask and backbone are showed in blue and white respectively. For the model-fitting approach (spherical and rod-like), the data and model are showed in green and magenta respectively. LF: long filamentous, SF: short filamentous, LS: large spherical, SS: small spherical, RD: rod-shape, UK: unknown. *L_F_*, *D_LS_*, *L_RD_* and *W_RD_* represent the length of the filamentous particles, the diameter of the large spherical, the length of the rod-shaped particles and the width of the rod-shaped particles respectively. Images of individual particles cover a field of view of 1.6 × 1.6 µm.

The images obtained with TIRF-SIM show a number of stereotypical virus structures in NDV samples labelled for HN, indicating a large morphological diversity in the virus populations that may stem from variability occurring during viral replication or at the purification stage. Understanding the origins and consequences of such heterogeneity informs not only on the life cycle of the virus but can also provide essential insights into the virus production process to manufacturers of virus-based therapeutics. An automated classification of virus shapes would enable the quantification of virus heterogeneity and permit further analysis of each individual class independently. The workflow to achieve these goals is shown in Figure 2.

**Figure 2—figure supplement 1.**
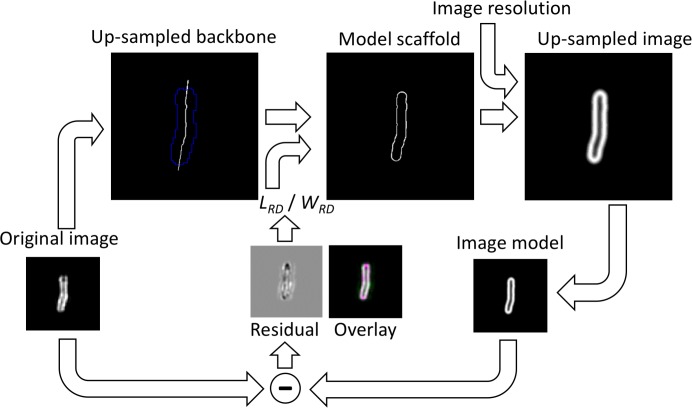
Image model for the analysis of the rod-shaped particles. The original image is segmented and thinned to obtain the backbone of the particle. The backbone is up-sampled and interpolated outside the particle. It is then used to compute the model scaffold. For this, each end of the backbone is independently grown or reduced to adjust its length, hence *L_RD_ = L_tot_ – L_1_ – L_2_*, where *L_tot_* is the maximum length of the extended backbone, and *L_1_* and *L_2_* are the adjusted distances by which the backbone length is adjusted on each end respectively. Then, the image is dilated by a disk-shaped kernel of radius equal to half *W_RD_*. The outline of this image gives the model scaffold. The scaffold is then convolved with a Gaussian kernel representing the effect of image resolution (here 90 nm) and the image is down-sampled again to the original image size. The optimal *L_RD_* and *W_RD_* are those that minimize the difference image and the *χ (Müller and Heilemann, 2013)*.

Individual virus particles are first identified by automated segmentation and then fed to the ML routine for classification. We used a supervised ML algorithm (here a random forest algorithm (Breiman, 2001)) to ensure the robustness of the method and for ease of implementation. We identified six major structural classes in the NDV samples which we divide into long and short filamentous, small and large spherical, rod-like and unknown structures. The unknown class is made of clumps of viral material with no consistent and identifiable shapes. A control sample that was prepared identically to the other samples except without virus particles present allowed us to identify that non-specific bindings of antibodies appear as rare, dim and small point-like structures that could easily be discriminated and excluded from further analysis.

The filamentous (long and short) class is further analysed by automatic extraction of the linear backbone of structures and measurement of their length. The width of these filamentous structures appeared to be limited by the resolution of the imaging technique (~90 nm for TIRF-SIM, see Figure 1—figure supplement 1, but also observed in higher resolution approaches such as *d*STORM) and therefore, we considered the filamentous class as 1D structures. The spherical structures were analysed by estimating their equivalent radius from the area of the particle. We note that other methods for estimation of the radius, for example the ellipsoid localization microscopy (ELM) analysis (Manetsberger et al., 2015), could also be used here. The latter fits a shape model to imaging data to permit the extraction of structural parameters with precision higher than the inherent resolution of the imaging method (Laine et al., 2015; Manetsberger et al., 2015). A similar model-based fitting approach was used to fit rod-like viral particles and to obtain length and width parameters for this structural class (see Materials and methods section and Figure 2—figure supplement 1 for details).

### Classification of virus structures using supervised machine learning algorithms

**Figure 3.**
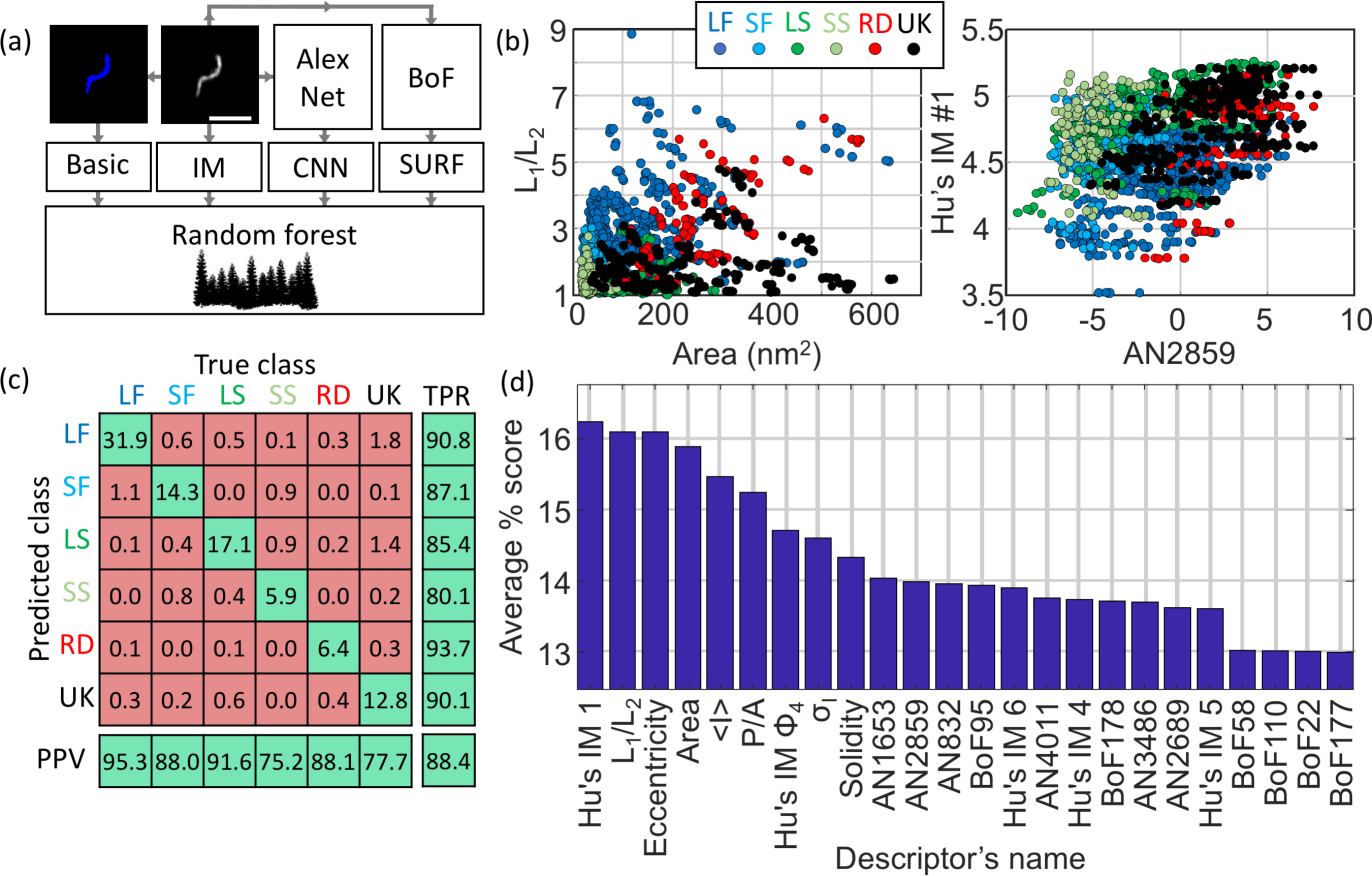
Machine learning-based classification. (**a**) Building the list of predictors from basic features, image moments, convolutional neural network (CNN) features and SURF bag of features (BoF). (**b**) Example of 2D scatter plots of pairs of predictors showing how some predictors allow identification of class clusters. (**c**) Confusion matrix obtained from the random forest showing the high true positive rate (TPR) and positive predictive values (PPV) of the classification. All numbers shown here are in percentage. (**d**) Scoring of the predictors sorted in descending order. IM: image moment. AN: AlexNet feature. BoF: SURF features. L_1_/L_2_: ratio of long axis over short axis. <I> : average intensity. P/A: perimeter to area ratio. σ_I_: standard deviation of intensity. LF: long filamentous, SF: short filamentous, LS: large spherical, SS: small spherical, RD: rod-shape, UK: unknown.

The structural classification was performed using a supervised ML algorithm which allows for rapid and automated classification of large datasets. The choice of algorithm and the set of features (often called predictors) extracted for each identified particle were optimised to maximise the overall accuracy of the model based on the training dataset (comprising of 370 manually annotated particles). Here, the model accuracy is defined as the fraction of correctly classified particles across all classes. Figure 3(a) describes the list of chosen individual features (selected from basic shapes features, Hu’s image moments (Hu, 1962), features obtained from the pre-trained convolutional neural network (CNN) AlexNet (Krizhevsky et al., 2012) and from Speeded Up Robust Features, SURF (Bay et al., 2008)). The predictors were selected based on the following criteria: basic structural features of the particles (e.g. area, eccentricity) and Hu’s moments were chosen because they are rotationally and translationally invariant. For the features from AlexNet and SURF a feature selection approach was designed based on maximising the standard deviation across the different structural classes. This approach constitutes a more rational choice compared to simple principal component analysis (PCA), which does not typically take the information regarding the classes into account, therefore our method selects for predictors that have high potential for class discrimination. This data reduction narrowed down the number of predictors to six for AlexNet and six for SURF.

**Figure 3—figure supplement 1.**
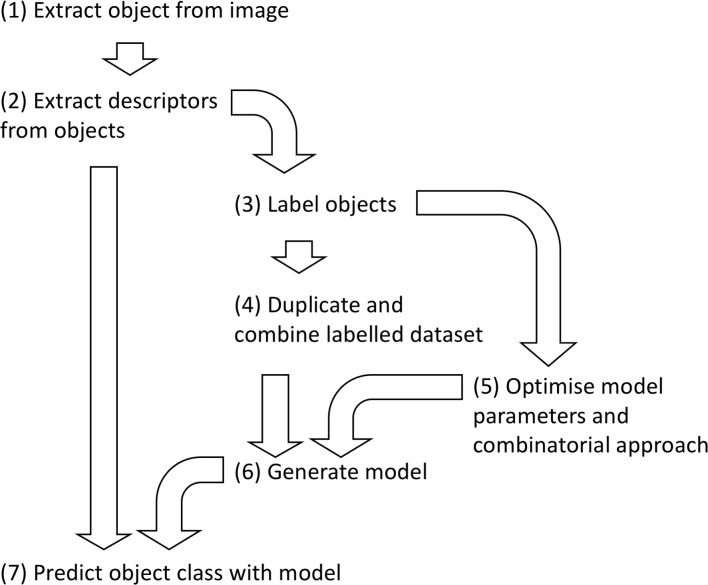
Flowchart describing the machine learning pipeline used here for image classification. A strong emphasis should be put on the choice of predictors and the quality of the manual annotation (training dataset) prior to classification, as this will largely determine the quality of the classification.

A total of 24 predictors was finally chosen: seven based on basic shapes (area, ratio of axis lengths, eccentricity, solidity, perimeter-to-area, mean intensity, standard deviation of pixel intensities), 5 of Hu’s image moments (Hu1, Hu4, Hu5, Hu6 and Phi4), six features obtained from the pre-trained convolutional neural network (CNN) AlexNet and six from a SURF bag of features. The classification workflow is described in Figure 3—figure supplement 1.

Panels described in Figure 3(b) show examples of scatter plot from arbitrarily chosen pairs of predictors highlighting that some predictors support classification across specific classes better than other combinations (identifiable clusters of certain classes). Here, the training dataset was used to build a scatter plot of pairs of predictors with the knowledge of their true classification (see colour scheme). For instance, the pair of predictors *L_1_/L_2_* and *Area* shows a good separation between long filamentous (dark blue labels) and unknown structures (black labels). The confusion matrix (Figure 3(c)) highlights the effective true positive rate (TPR) and positive predictive values (PPV) across the different classes with a model accuracy of 88.4%. We note that some long filamentous viruses are misclassified as small filamentous, some small filamentous are misclassified as small spherical and that, on occasion, some unknown structures populate the predicted long and large spherical structure classes. Considering the simple shapes of these viruses, it is expected that a small fraction of particles are misclassified as structures with close resemblance.

The scoring of the predictors presented in Figure 3(d) indicates the average accuracy of each individual predictor. A high score indicates a high capacity to discriminate between different classes. The scoring was performed by measuring the accuracy of the classification for many combinations of predictors and distributing the accuracy score across the predictors tested (see Materials and methods for details). In other words, if a combination of 2 predictors alone give an accuracy of 60%, a score of 30% is awarded to both individual predictors. This method was repeated and scores represent averages across >13,000 different combinations of predictors.

### Structural details of an NDV virus population

**Figure 4.**
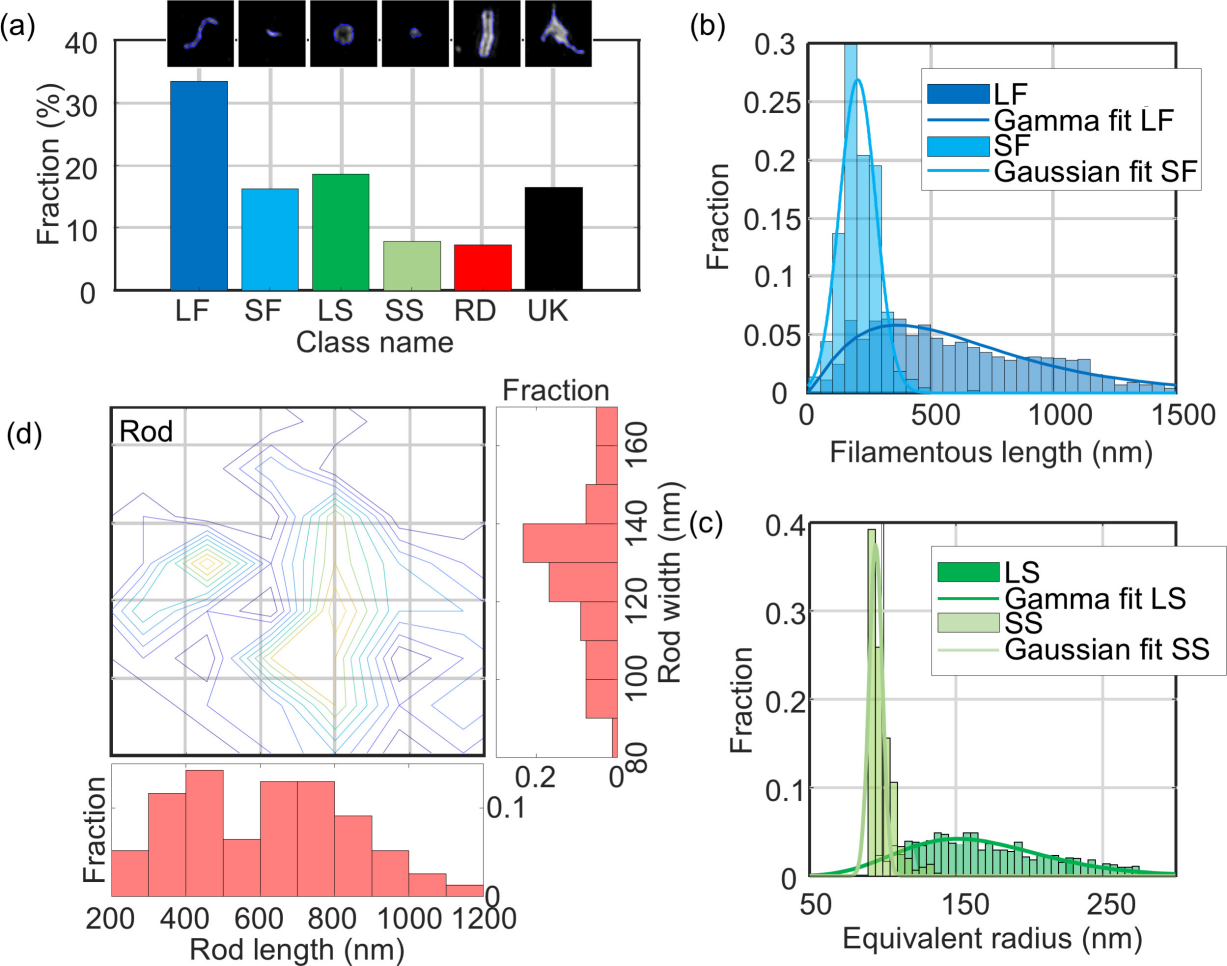
Quantitative analysis of NDV. The distribution of structural parameters for all classes was obtained from a total of ~6500 virus particles. LF: long filamentous, SF: short filamentous, LS: large spherical, SS: small spherical, RD: rod-shape, UK: unknown. Images of individual particles cover a field of view of 1.6 × 1.6 µm. 10.7554/eLife.40183.013Figure 4—source data 1.Source data for Figure 4.

We analysed a total of ~6500 particles using MiLeSIM and established that 49.7% of NDV particles presented a filamentous shape whereas the large spherical, small spherical and rods represent 18.6%, 7.8% and 7.3% of the total population, respectively (Figure 4(a)). In addition to structural classification, the high-resolution images also permitted a dimensional analysis to be performed at the single particle level. We estimated the particle radius from both small and large spherical particles by calculating the equivalent radius from the particle area; backbone extraction to the short and long filamentous particles, to estimate the particle length; and designed a model fitting for the rod structures. Figure 4 shows the distribution of structural parameters for each class. We observe that both long filamentous and large spherical are well described by a Gamma distribution whereas the small filamentous and small spherical are well described by a Gaussian distribution. The model-fitting applied to the rod-shaped particles (see Materials and methods and Figure 2—figure supplement 1 for details) allows the extraction of both the width and length of each particle. Therefore, it is possible to plot the distribution of structural parameters as a contour plot Figure 4(c)).

**Figure 4—figure supplement 1.**
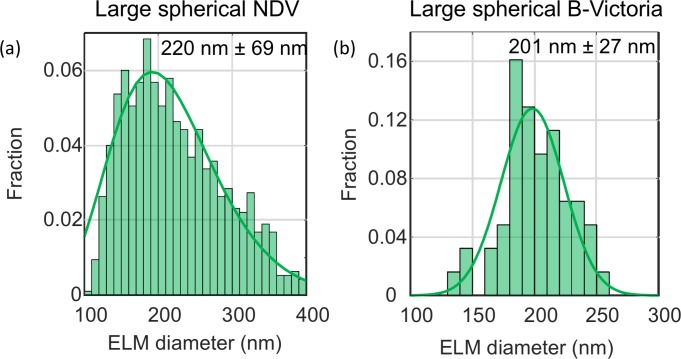
ELM analysis of the large spherical NDV. (**a**) and B-Victoria LAIV (**b**) viruses. The distribution of diameters were fitted to a Gamma and Gaussian distributions respectively. The mean diameters and standard deviations of the data are shown.

We estimated the mean and standard deviation of the structural parameters from the distributions and obtained: *L_LF_* = 650 ± 430 nm, *L_SF_* = 200 ± 100 nm, *D_LS_* = 338 ± 94 nm, *D_SS_* = 190 ± 10 nm. For the rod-shaped particles, we observed that the width *W_RD_* = 135 ± 30 nm and the length *L_RD_* = 610 ± 350 nm (all rounded to two significant figures, ± represents the standard deviation of the distribution). These values are distributed around two populations as shown on the contour plot in Figure 4(d).

However, we note that the radius analysis based on the area of the particle used here constitutes an overestimate of the physical radius of the particle due to the broadening caused by the point-spread function. It is possible to estimate a more accurate diameter of the underlying spherical structures by using the ELM analysis. The results obtained from the ELM analysis of the large spherical structures are shown in Figure 4—figure supplement 1(a). The ELM diameter obtained for the large spherical particles (220 ± 69 nm) is in good agreement with an area-based diameter of 338 nm and a resolution of 90 nm.

It should be noted that the small spherical distribution is centred on the value of optical resolution of our SIM microscope, which indicates that the small spherical structures the small spherical structures are smaller than the point-spread-function.

### MiLeSIM is capable of assaying influenza strains used for vaccine production in purified and non-purified samples from the production line

**Figure 5.**
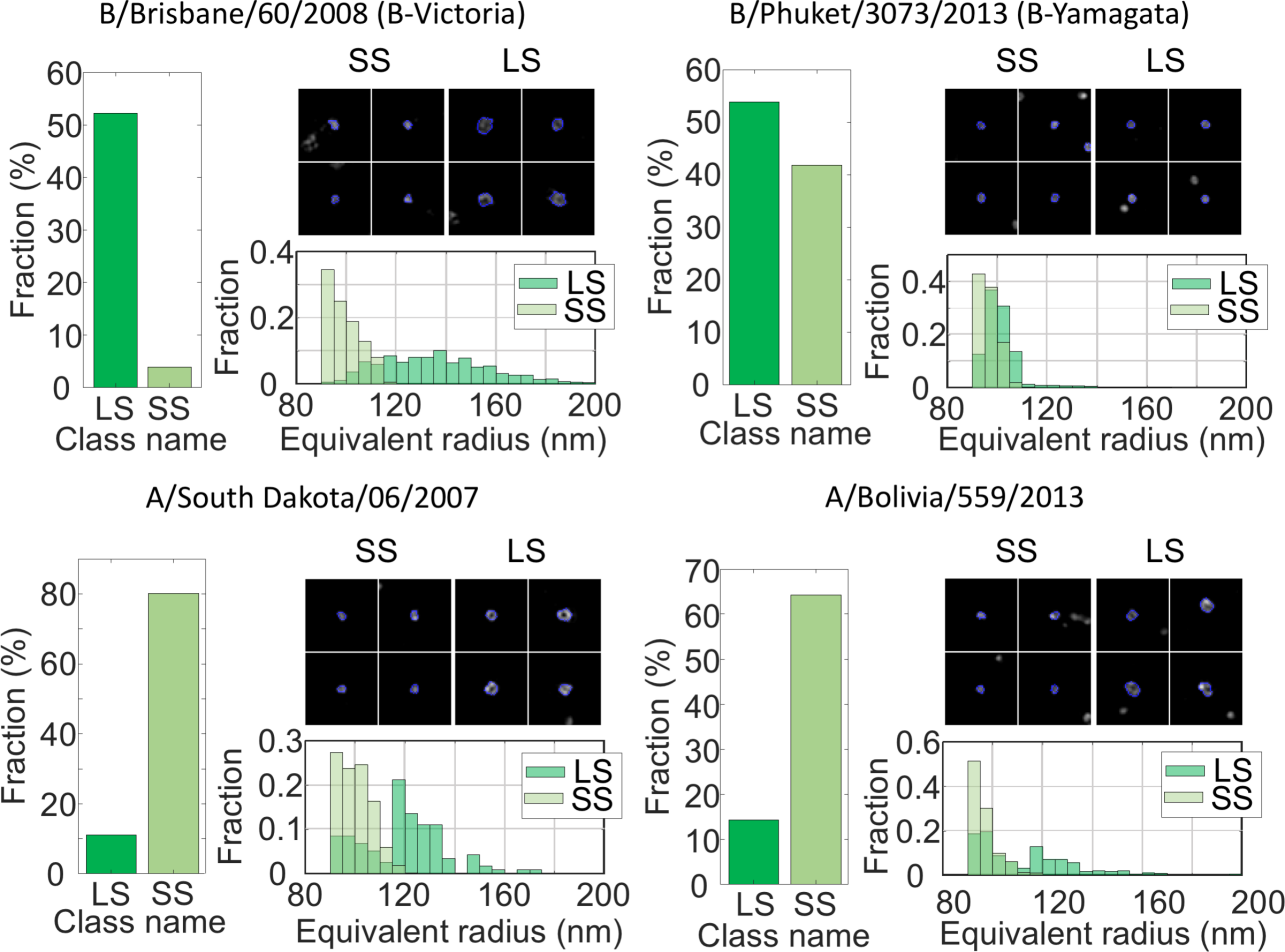
MiLeSIM approach applied to Live Attenuated Influenza Virus (LAIV). 2 types of B and A viruses were analysed here. The population was dominated by small and large spherical particles. The distributions of equivalent radius are shown here for both the large and small spherical for direct comparisons. The number of particles analysed were N = 3,821, 4704, 1062 and 1756 for B/Brisbane/60/2008 (B-Victoria), B/Phuket/3073/2013 (B-Yamagata), A/South Dakota/06/2007 and A/Bolivia/559/2013 respectively. Images of individual particles cover a field of view of 1.6 × 1.6 µm. 10.7554/eLife.40183.016Figure 5—source data 1.Source data for Figure 5.

We applied our approach to four different strains of Live Attenuated Influenza Virus (LAIV) immuno-labelled for the glycoprotein Hemagglutinin (HA) present on the exterior of the viral envelope. The shape of the virus particles obtained here were classified using the same classifier as for NDV. The LAIV virus population was dominated by spherical structures (>60%). Figure 5 shows the distribution of particle sizes for four virus strains: a B-Victoria subtype (B/Brisbane/60/2008), a B-Yamagata subtype (B/Phuket/3073/2013) and two subtype A H1N1 strains (A/South Dakota/06/07 and A/Bolivia/559/2013). The fractions of small and large spherical particles are shown, as well as the equivalent radii and representative images of the viruses. It is clear that B-Victoria particles consist of mostly large hollow particles with an equivalent radius of ~130 nm, a value that is in good agreement with the ELM analysis and a resolution of 90 nm (Figure 4—figure supplement 1(b)).

**Figure 5—figure supplement 1.**
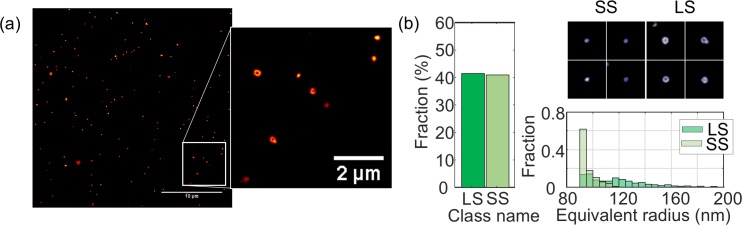
Structural analysis of B-Victoria LAIV obtained from pool harvested fluid (PHF). (**a**) TIRF-SIM images. The images acquired here using PHF show an identical image quality as with highly purified samples. (**b**) Structural analysis of N = 1295 virus particles. Images of individual particles cover a field of view of 1.6 × 1.6 µm.

In contrast, the B-Yamagata strain shows small and large particles of equal amount, indicating that the particles sizes are distributed around the region of overlap between small and large particles. This is confirmed by the nearly identical equivalent radius distributions.

Both A strains appeared clearly dominated by small spherical particles with sizes close to the resolution limit of our imaging. However, our high-throughput approach reveals subtle differences in the distribution of small spherical structures where the A/South Dakota viruses appear more heterogeneous (standard deviation ~10 nm), whereas the A/Bolivia viruses are sharply distributed (standard deviation ~4 nm).

We also investigated the potential of directly imaging pool harvested fluid (PHF). LAIV are commonly propagated in embryonated hens’ eggs where progeny viruses are released into the allantoic fluid of the egg. This fluid is harvested from numerous eggs and pooled. This constitutes a very basic and commonly used virus material. It is easy to produce and does not undergo any downstream purification. Consequently, PHF is impure, containing a variety of egg-derived impurities. The high molecular specificity of fluorescence microscopy allowed us to visualize the structure of the viruses with the same image quality directly in PHF despite the presence of a large amount of impurities (Figure 5—figure supplement 1(a)). The structural analysis of the B-Victoria strains from MVB (Figure 5) and PHF (Figure 5—figure supplement 1(b)) allows us to decipher the effect of purifications steps on the structural properties of the population. The fraction of unknown structures dropped from 23% to 8% between MVB and PHF respectively. This change in fraction of unknown structure may be a result of the different densities of viruses on the cover slip. We observed a lower density of virus particles in the PHF preparations, which may lead to fewer aggregated classes and therefore fewer unidentifiable structures. In addition, whereas the PHF shows a nearly equal amount of small and large spherical structures, the MVB preparation is missing a large population of small spherical compared to the PHF. This is also reflected by the larger average diameters observed in the MVB compared to the PHF (*D_SS_* = 191 ± 12 nm and 198 ± 14 nm and *D_LS_* = 241 ± 56 nm and 275 ± 49 nm for PHF and MVB respectively).

MiLeSIM therefore enables the study of unpurified samples and allows probing the virus production at any intermediate levels of production and purification. This constitutes a strong advantage over EMtechniques which require the use of highly purified samples and elaborate preparation protocols.

## Discussion

We have demonstrated the potential of high-throughput imaging of virus structures, taking advantage of the optimal combination of speed and resolution afforded by the TIRF-SIM imaging method. TIRF-SIM provided sufficient resolution to identify, discriminate and analyse individual viral structural classes with high specificity, even in non-purified samples. Our approach combines machine learning to classify NDV viruses, followed by a model-based or direct quantification of virus structural parameters. The method yielded similar results both in purified samples and in samples from unfiltered PHF offering promise for use as an assay during virus production. We were able to image up to ~220 particles/second at 90 nm resolution, vastly increasing imaging throughput compared to alternative super-resolution methods, improving sensitivity and specificity in comparison to EM. Furthermore EM does not feature the specificity to analyse virus samples in their aqueous, unaltered unpurified forms. We were able to observe large structural variabilities in the NDV population and also between different strains of LAIV.

Our particular classification uses random forest with a selection of predictors from simple shape parameters, rotational and translational invariant image moments and features from AlexNet and common feature for image recognition such as SURF. The model accuracy is ~88.4% and the mis-classifications occur between classes that are similar (between small spherical and small filamentous for instance). The structural parameters that we extract from the model fitting are precise beyond the image resolution as they take into account the finite optical resolution. This therefore reveals subtle differences in populations such as the two sub-classes observed in the rod-shaped class. This approach will be beneficial especially when heterogeneous populations are present and need to be quantified. In future, such information can be correlated with functional characteristics of produced virus classes and production parameters can accordingly be optimised. The approach thus holds great promise for the production of virus-based therapeutics. We note, however, that the methods presented are generally applicable to other systems and they are not restricted to a particular type of fluorescence microscopy, SRM or not.

## Materials and methods

### Sample preparation

The purified NDV samples were prepared on cover slips as previously described (Laine et al., 2015). Briefly, viruses were adhered on poly-L-lysine-coated Ibidi 8-well dishes, fixed, permeabilised and immuno-labelled for the envelope glycoprotein Hemagglutinin-Neuraminidase (HN) with primary antibodies (mouse anti-hemagglutinin-neuraminidase HN, Abcam, UK) followed by secondary labelling (goat anti-mouse labelled with Alexa Fluor 647 for *d*STORM, with Alexa Fluor 488 for TIRF-SIM and with ATTO647-N for STED, Abcam, UK).

The LAIV samples were prepared identically but using primary antibodies originating from MedImmune in-house, non-commercially available monoclonals that target the viral glycoprotein Hemagglutinin (HA) present on the exterior of the viral envelope: F16 mouse antibody for B-Victoria, Infa0121 mouse antibody for B-Yamagata and FY1 human antibody (Kallewaard et al., 2016) for A South Dakota and A Bolivia. The corresponding secondary antibodies were used (donkey anti-mouse DyLight 488 labelled or rabbit anti-human DyLight 488 labelled antibodies, ThermoFisher). All virus samples originated from the monovalent bulk (MVB) and are therefore highly purified, unless indicated in the text, where the direct pool harvest fluid (PHF) was used.

### TIRF-SIM, STED and *d*STORM imaging

Our custom-built TIRF-SIM system was described previously (Young et al., 2016). We used an Olympus UAPON 100x TIRF NA = 1.49 and an Orca Flash 4.0 camera, with a sample pixel size of 64 nm. A total of 9 SIM images were acquired (three phases, three orientations) with a camera exposure time of 200 ms and ~250 µW of 488 nm laser, measured at the back aperture of the objective. The SIM images were obtained using the reconstruction code provided by Dr Lin Shao (Shao et al., 2011), providing images with doubled resolution and 32 nm final pixel size using a Wiener filter of 0.01. The STED imaging was performed on our custom-built STED microscope as described previously (Mahou et al., 2015). The *d*STORM imaging was performed on a custom-built single-molecule microscope previously described (Ströhl et al., 2017; Wong et al., 2017) and with mercaptoethylamine (MEA) buffer as previously described (Laine et al., 2015). The *d*STORM image reconstruction was carried out using rapidSTORM 3 (Wolter et al., 2012).

The resolution achieved by the TIRF-SIM microscope was assessed by identifying the edge of the spatial frequency support using the SIMcheck plugin (Ball et al., 2015), as shown in Figure 1—figure supplement 1. For STED microscopy, the resolution was estimated from cross-sections of 20 nm beads and reporting the full width at half maximum (FWHM). The *d*STORM resolution reported here was obtained from the FWHM of the localization precision, estimated by (Thompson et al., 2002).

### Classification

All segmentations, predictors extractions and classifications were performed using MATLAB (Mathworks). The code is freely available (Laine, 2018). A general diagram of the method is shown in Figure 1—figure supplement 1. The segmentation was obtained by an initial Otsu binarization and refined by active contour. This allowed a better outline of the particles and efficient separation of particles in close proximity. The particles that were judged too small or too dim to be real particles (based on criteria obtained from the control sample) were excluded from further analysis.

The basic shape features were extracted using the MATLAB function *regionprops*. Hu’s image moments were computed from the 71 × 71 pixels particle image centred on the centre of mass of the particle. The absolute values of the logarithm of the moments were used in the classification. For the features obtained from AlexNet (Krizhevsky et al., 2012), the individual 71 × 71 pixels images were resized to 227 × 227 pixels and used as all three color layers of the RGB images taken by AlexNet. Then, feature extraction was performed using AlexNet as a pre-trained network. 4096 features were obtained and data reduction was performed to limit the number of predictors used. For this, the features were averaged within each individual class and the standard deviation of every feature across the classes was computed. The six features with the highest standard deviation was selected. For the SURF features, first a bag of visual words was created from the training dataset, this bag was then used to check the presence of visual words in the 71 × 71 pixels images of individual particles. Similarly to AlexNet features, we selected only the six visual word features with the highest standard deviation across the different classes for classification. This allowed the computation of a total of 24 features for ML.

The classification was performed using a random forest algorithm. The training dataset was made of 370 manually labelled individual particles and was used to train the random forest across 60 epochs. The classification was validated by 10-fold cross validation on the same dataset. The confusion matrix obtained from this cross-validation is shown in Figure 3. At the training stage, the training dataset was augmented 5-fold by transforming the images with image translation and rotation randomly picked between 0 and 1 pixel and between 0 and 360 degrees respectively.

The accuracy of the model was estimated by calculating the fraction of correctly classified particles across all classes.

$$accuracy=\frac{Numberofparticlescorrectlyclassified}{Totalnumberofparticles}$$

### Predictor scoring

The predictors were scored by computing the accuracy of the random forest trained on the training dataset but with only subsets of features. Out of the 24 predictors all combinations of 2, 3, 4, 24, 23 and 22 predictors were tested corresponding to a total of 13,227 combinations of predictors. For each combination of predictors, the accuracy obtained was split equally across the different predictors used, producing a ‘local’ accuracy for each feature. This local score was average across all combinations using a specific feature to obtain the global score.

$$S_{i}=\frac{1}{N_{c}^{i}}\sum_{j=1}^{N_{c}}a_{ij}\frac{P_{j}}{n_{j}}$$

Where *S_i_* is the global score of the feature *i*, *N_c_^i^* is the total number of combinations tested involving feature *i*, *a_ij_* is a factor reflecting the presence of the feature *i* in the combination *j. a_ij_* is equal to one if *i* is present in *j*, 0 otherwise. *P_j_* is the accuracy of the combination *j*, *N_c_* is the total number of combination tested and *n_j_* is the number of features present in the combination *j*.

### Quantitative analysis

All quantitative analyses were performed using MATLAB (Mathworks). The code is freely available (Laine, 2018). The length of the filamentous structures were extracted by measuring the geodesic distance along the skeletonized image of the filament. The ELM analysis is freely available (Manetsberger et al., 2015) and the code was adapted to insert within the workflow of our approach. For ELM analysis, we observed no significant ellipticity in the spherical virus particles and fitted spherical shapes to extract the radius of the particles (Figure 4—figure supplement 1).

The equivalent radius *r* of the spherical particles were simply calculated from the area *A* of the segmented particle.

$$r=\sqrt{\frac{A}{\pi }}$$

The image model for the rod-shaped particles is presented in Figure 1—figure supplement 1. Briefly, the backbone of the particle was extracted by image thinning and then dilated by a disk-shaped kernel of radius equal to half of the width of the rod. The length of the rod could be adjusted by shortening the ends of the backbone or by extrapolating it outwards to lengthen it. The interior pixels of the image obtained were removed to leave the outline of the particle shape. This outline was then convolved with a Gaussian kernel in order to take into account the effect of the image resolution (here 90 nm). The intensity, the width and length of the model image were adjusted to minimize the sum of the square difference of intensity *χ^2^*.

$$\chi^{2}=\sum_{ij}{\left[I_{m}\left(i,j\right)-I_{d}(i,j)\right]}^{2}$$

Where *i* and *j* refer to the indices in the image, *I_m_(i,j)* is the image model, and *I_d_(i,j)* is the data image.

### Supplementary Note 1: Throughput of the method

The imaging throughput of the method can be assessed in terms of number of particles imaged per second. The field-of-view achievable in our TIRF-SIM system is ~32 µm x 32 µm and a high quality sample preparation can yield a virus particle density of ~1 particle/µm^2^. Therefore, with an acquisition time of 200 ms/SIM raw frame (with a total of 9 frames), we assess that our single frame particle throughput can reach ~500 imaged particles/s. However, the acquisition of two consecutive fields-of-view are affected by imaging dead time as a consequence of stage movement and refocussing. In the study presented here, this step was done manually and took approximately 2–3 s. Therefore, a practical throughput achievable for the imaging is of the order of ~220 particles/s. We note however that both acquisition times and the stage movement time can be easily reduced by increasing illumination power and automation respectively. This makes the 500 particles/s not an unreasonable estimation for the achievable throughput of a further optimised acquisition. The throughput of the method can also be regarded as the time necessary to perform the complete study from sample preparation to analysis. Table 2 indicates typical times necessary to perform the individual steps of the workflow. This table indicates that a full structural analysis of a particular sample can be obtained within a day.

**Table 2.** Estimation of the time necessary to perform individual steps involved in MiLeSIM. Sample preparation was estimated based on standard immuno-labelling protocols. The computational times were assessed on an analysis machine with an i7 processor at 3.5 GHz and 64 GB of RAM.

Step	Description	Time
Sample preparation	Plating, permeabilising and immune-labelling of virus particles	2–3 hr
Instrument set-up	Quality check of set-up alignment, calibration and sample mounting	30 min
Imaging	Image acquisition, stage movement and refocus for ~ 50,000 particles (50 fields-of-view)	30 min
SIM reconstruction	SR reconstruction of 50 fields-of-view	<30 min
Classification on unknown data	Extraction of predictors and classification (for 50 fields-of view)	1h
Structural analysis	Extraction of structural parameters for each classes (for 50 fields-of view)	1h
Data curation for training dataset	Generating manually labelled particle dataset (performed only once) for ~ 500 particles	2h
Generating classification model	Data augmentation, extraction of predictors, training of the model, cross-validation on ~ 500 particles (performed only once)	5–6 hr

## Additional files

Transparent reporting form

### Data availability

Analysis code for image segmentation, machine learning training, classification and structural analysis is available via GitHub (https://github.com/Romain-Laine/MiLeSIM; copy archived at https://github.com/elifesciences-publications/MiLeSIM). Source data files have been provided for Figures 4 and 5.
