# Supplementary material for: Structured illumination microscopy combined with machine learning enables the high throughput analysis and classification of virus structure
Source: eLife. 2018 Dec 13;7:e40183. doi: 10.7554/eLife.40183 (PMC6331195; doi:10.7554/eLife.40183)
Supplement: Peer review history

# Decision letter

**DOI:** 10.7554/eLife.40183.020

**Affiliations:** University of WollongongAustralia; University of LeuvenBelgium

In the interests of transparency, eLife includes the editorial decision letter and accompanying author responses. A lightly edited version of the letter sent to the authors after peer review is shown, indicating the most substantive concerns; minor comments are not usually included.

Thank you for submitting your article "Structured illumination microscopy combined with machine learning for the high throughput analysis of virus structure" for consideration by *eLife*. Your article has been reviewed by three peer reviewers, one of whom is a member of our Board of Reviewing Editors, and the evaluation has been overseen by Arup Chakraborty as the Senior Editor. The following individual involved in review of your submission has agreed to reveal his identity: Peter Dedecker (Reviewer #2).

The reviewers have discussed the reviews with one another and the Reviewing Editor has drafted this decision to help you prepare a revised submission. We hope you will be able to submit the revised version within two months.

Summary:

The authors describe the use of TIRF-SIM to visualize structural heterogeneity within populations of viral particles and use machine-learning approaches to classify the different morphologies. With the speed of data acquisition and the automated classification, the method is an interesting approach to introduce robust and fast quality control of production of viral particles in an industrial setting (e.g. vaccine production).

Essential revisions:

1) Since this manuscript is a submission for the Tools and Resources section of the journal, there is less of a requirement for new mechanistic insight, but an essential need for a clear justification of why the methodology will be better than what is currently used. The reviewers commented on a lack of clear justification of the work and the need to place it in context of methods that are currently used to characterise morphologies in heterogeneous populations of viral particles. Importantly, the work should provide a quantitative analysis of how the described workflow performs compared to established workflows (the authors mention electron microscopy).

2) Also, the manuscript lacks detail in many places to truly understand what the authors did and in what order.

Not until the Materials and methods does it become clear what was being imaged in the paper, and even then, a description of the primary antibodies is not very informative with respect to what structures are being identified (envelope, capsid…?). A brief mention of what is being measured early would greatly improve the readability of the paper.

More details regarding the high throughput nature of the measurements are needed. How do the authors achieve the 500 particles/second? Do they have 1000 particles in a field of view? (1000 particles/1.8 s data collection time). How long does it take to switch between fields of view? How long does the software need to extract the 500 super resolution particles? The authors should also mention how long the analysis takes (both for training and analyzing the data with the trained algorithm).

3) The described method for feature selection hinges on the evaluation of their predictive power, though no clear definition is provided of how this is calculated.

The main issue is with Figure 4C, showing the radii of large and small spherical particles. First of all, LS and SS both seem to have the same average radius. Why? The authors also remark that "It may first appear surprising that the distribution of radius of small spherical lies within that of the large spherical but in the ELM analysis the broadening of the image structure due to the finite optical resolution is effectively removed by taking into account the point spread function (PSF)." We don't understand this argument. Consider also that the example SS and LS images (e.g. Figure 4A) are clearly different in size. Are these images then not representative?

In the next sentence, the authors argue that the SS distribution is centered on the optical resolution of the instrument, suggesting that the SS particles are effectively point-like. First of all, the LS distribution is also centered on this value, so the conclusion is that these are also point-like? Second of all, the ELM analysis is supposed to correct for this, judging by the immediately preceding sentence? It is unclear what the authors are trying to argue here.

4) When analyzing the viruses in the pool harvested fluid, the authors should quantify the percentage of unknown objects compared to measurements with purified samples. The authors could also use their method to see what impact purification has on the distribution of structures in the sample.

5) Figure 4. Extraction of the size distribution is an interesting aspect of this work. However, it is problematic that the radius of the larger spheres is often smaller than the small spheres. Particularly for spherical particles, it is possible to determine radii with better resolution than the resolution limit when using deconvolution especially for the virus, which is on the size scale of the PSF. If it is really a point-like structure, this suggest that you are not measuring viruses. Could it be unspecific binding of the secondary antibodies to the surface or individual proteins that are not associated to the viruses in this class?

# Reply: Author response

**DOI:** 10.7554/eLife.40183.021

Essential revisions:

1) Since this manuscript is a submission for the Tools and Resources section of the journal, there is less of a requirement for new mechanistic insight, but an essential need for a clear justification of why the methodology will be better than what is currently used. The reviewers commented on a lack of clear justification of the work and the need to place it in context of methods that are currently used to characterise morphologies in heterogeneous populations of viral particles. Importantly, the work should provide a quantitative analysis of how the described workflow performs compared to established workflows (the authors mention electron microscopy).

We have now included a comparison between electron microscopy and our approach with a number of comments throughout the text and have also included a table summarising important performance parameters of the two methods (Table 1).

“Typically, this is achieved by extracting batches from the production process, with elaborate subsequent purification and preparation steps before characterisation by Transmission Electron Microscopy (TEM) (Gad, 2007; Goldsmith and Miller 2009; Brenner and Horne, 1959. […] It is therefore challenging for TEM to be of practical use during production operations.”

“Traditionally, EM has been the method of choice for observing sub-diffraction structures of virus particles (see Figure 1—figure supplement 2 for examples of particles). […] Also, the capability of investigating unpurified and aqueous samples makes TIRF-SIM ideally suited to the present application.”

“We were able to image up to ~220 particles/second at 90 nm resolution, vastly increasing imaging throughput compared to alternative super-resolution methods, improving sensitivity and specificity in comparison to TEM. Furthermore EM does not feature the specificity to analyse virus samples in their aqueous, unaltered unpurified forms.”

2) Also, the manuscript lacks detail in many places to truly understand what the authors did and in what order.

Not until the Materials and methods does it become clear what was being imaged in the paper, and even then, a description of the primary antibodies is not very informative with respect to what structures are being identified (envelope, capsid…?). A brief mention of what is being measured early would greatly improve the readability of the paper.

We have worked on the main text to improve clarity and flow of information. Regarding antibodies, NDV viruses were labelled by targeting Hemagglutinin-Neuraminidase (HN) via primary/secondary antibody labelling. HN is a viral envelope glycoprotein and therefore identifies the outline and overall shape of the virus particles. Additional details were added to the Results and Materials and methods sections.

“NDV viruses were labelled for the envelope glycoprotein Hemagglutinin-Neuraminidase (HN) and imaged with all three SRM imaging techniques (see Figure 1). […] Typical shapes observed with TIRF-SIM are shown in Figure 1B. TIRF-SIM provides clear structural details to discern filamentous, spherical and rod-like structures in large NDV populations.”

“The images obtained with TIRF-SIM show a number of stereotypical virus structures in NDV samples labelled for HN, […]”

For influenza, we would like to clarify that primary antibodies were all MedImmune inhouse, non-commercially available monoclonals that target the viral glycoprotein Hemagglutinin (HA) present on the exterior of the viral envelope. Additional details were added to the Results and Materials and methods sections.

“We applied our approach to four different strains of Live Attenuated Influenza Virus (LAIV) immuno-labelled for the glycoprotein Hemagglutinin (HA) present on the exterior of the viral envelope. The shape of the virus particles

obtained here were classified using the same classifier as for NDV.”

More details regarding the high throughput nature of the measurements are needed. How do the authors achieve the 500 particles/second? Do they have 1000 particles in a field of view? (1000 particles/1.8 s data collection time). How long does it take to switch between fields of view? How long does the software need to extract the 500 super resolution particles? The authors should also mention how long the analysis takes (both for training and analyzing the data with the trained algorithm).

We agree that aspects of imaging throughput should have been presented in more detail. We have now added a discussion on throughput achievable with our method in a supplementary note (Supplementary Note 1). The value originally quoted as 500 particles/s was based on an estimation of the maximal achievable throughput for the imaging conditions presented in the manuscript. As the reviewers point out, this estimation is based on ~1000 / field-of-view and a ~2s acquisition, conditions that are readily achievable in practice. In the examples shown, the microscope stage was moved and the field refocused manually, which took an average of ~2-3s. This therefore brings the actual imaging throughput to ~ 220 particles/s, which is what we now quote in the manuscript. However this value can be easily improved on through a reduction of acquisition times and automation of stage movement. This makes the 500 particles/s not an unreasonable estimation for a possible throughput.

Supplementary Note 1 also includes estimations of the time necessary for the different steps of the workflow proposed here.

3) The described method for feature selection hinges on the evaluation of their predictive power, though no clear definition is provided of how this is calculated.

Here, we defined the predictive power as the accuracy of the model. This describes the fraction of correctly classified particles, which corresponds to the sum of the correctly predicted fractions in the confusion matrix shown in Figure 3C. We now name our metric “accuracy” (instead of predictive power) and give a definition at its first instance.

“The choice of algorithm and the set of features (often called predictors) extracted for each identified particle were optimised to maximise the overall accuracy of the model based on the training dataset (comprising of 370 manually annotated particles). Here, the model accuracy is defined as the fraction of correctly classified particles across all classes.”

We also explicitly define what we mean by accuracy in the Materials and methods section.

“The accuracy of the model was estimated by calculating the fraction of correctly classified particles across all classes.

accuracy=NumberofparticlescorrectlyclassifiedTotalnumberofparticles

The main issue is with Figure 4C, showing the radii of large and small spherical particles. First of all, LS and SS both seem to have the same average radius. Why? The authors also remark that "It may first appear surprising that the distribution of radius of small spherical lies within that of the large spherical but in the ELM analysis the broadening of the image structure due to the finite optical resolution is effectively removed by taking into account the point spread function (PSF)." We don't understand this argument. Consider also that the example SS and LS images (e.g. Figure 4A) are clearly different in size. Are these images then not representative?

We agree that the way we presented the results for the spherical structures was confusing. The confusion came from extracting the radii for large and small spherical particles using different analysis methods and plotting them on the same graph. In the original manuscript, the radius obtained for the small spherical was extracted from an area analysis whereas the radius shown for the large spherical originated from the ELM analysis. The radii obtained by these 2 methods represent two different structural parameter for the images.

We have therefore changed Figure 4 to show only radius estimation by area analysis for both small and large spherical particles in Figure 4C.

The average diameters are now clearly separated: D_LS_ = 338 ± 94 nm, D_SS_ = 190 ± 10 nm.

We also added the following clarifications.

“The spherical structures were analysed by estimating their equivalent radius from the area of the particle. We note that other methods for estimation of the radius, e.g. the ellipsoid localization microscopy (ELM) analysis (Manetsberger et al., 2015), could also be used here.”

And the confusing section has now been deleted.

The ELM analysis now appears in Figure 4—figure supplement 1A and is introduced in the main text as the following paragraph.

“However, we note that the radius analysis based on the area of the particle used here constitutes an overestimate of the physical radius of the particle due to the broadening caused by the point-spread function. […] The ELM diameter obtained for the large spherical particles (220 nm ± 69 nm) is in good agreement with an area-based diameter of 338 nm and a resolution of 90 nm.”

In the next sentence, the authors argue that the SS distribution is centered on the optical resolution of the instrument, suggesting that the SS particles are effectively point-like. First of all, the LS distribution is also centered on this value, so the conclusion is that these are also point-like? Second of all, the ELM analysis is supposed to correct for this, judging by the immediately preceding sentence? It is unclear what the authors are trying to argue here.

We hope that the amended presentation of the data addresses this point clearly. By using the equivalent (area-based) radius analysis for both small and large spherical particles, the distinction between the two classes is now clear, see the amended Figure 4.

The ELM analysis is now presented as an alternative analysis for the estimation of the radius in Figure 4—figure supplement 1. See the comment above and comment 5) below for further discussion on ELM.

4) When analyzing the viruses in the pool harvested fluid, the authors should quantify the percentage of unknown objects compared to measurements with purified samples. The authors could also use their method to see what impact purification has on the distribution of structures in the sample.

We now present the results of the structural analysis of B-Victoria from PHF in Figure 5—figure supplement 1B and describe our observations in terms of unknown objects and changes in distribution of structures.

“The high molecular specificity of fluorescence microscopy allowed us to visualize the structure of the viruses with the same image quality directly in PHF despite the presence of a large amount of impurities (Figure 5—figure supplement 1A). […] This is also reflected by the larger average diameters observed in the MVB compared to the PHF (*D_SS_* = 191 ± 12 nm and 198 ± 14 nm and *D_LS_* = 241 ± 56 nm and 275 ± 49 nm for PHF and MVB respectively).”

*5) Figure 4. Extraction of the size distribution is an interesting aspect of this work. However, it is problematic that the radius of the larger spheres is often smaller than the small spheres. Particularly for spherical particles, it is possible to determine radii with better resolution than the resolution limit when using deconvolution especially for the virus, which is on the size scale of the PSF. If it is really a point-like structure, this suggest that you are not measuring viruses. Could it be unspecific binding of the secondary antibodies to the surface or individual proteins that are not associated to the viruses in this class?*

With the amended analysis, now using equivalent diameter for both large and small spherical particles, the two classes are clearly discriminated and, as expected, the radii of large spherical particles are distributed around a range of radii larger than those of the small spherical particles (see amended Figure 4C). The reviewers mention the possibility to extract radius with better precision than the resolution limit. This is the intention with the ELM analysis, which takes the PSF into account (to some extent in a similar way to a deconvolution operation, but here based on a parametric shape modelling of the sample). The ELM analysis is now shown in Figure 4—figure supplement 1 for both the large spherical of NDV and that of B-Victoria.

As for unspecific binding, we excluded this possibility of this by measuring a control sample (sample prepared identically to the virus samples but in the absence of virus particles) and having a thresholding step prior to further analysis to exclude background signal. This point was made clearer in the main text:

“A control sample that was prepared identically to the other samples except without virus particles present allowed us to identify that non-specific bindings of antibodies appear as rare, dim and small point-like structures that could easily be discriminated and excluded from further analysis.”

As well as in the Materials and methods:

“The particles that were judged too small or too dim to be real particles (based on criteria obtained from the control sample) were excluded from further analysis.”
